# Association of alkaline-phosphatase/albumin ratio with all-cause mortality in critically ill patients with ischemic stroke: a retrospective study

**DOI:** 10.3389/fneur.2025.1567767

**Published:** 2025-04-30

**Authors:** Tao Zheng, Mengmeng Guo, Yating Han, Guanglu Li, Xianhua Wang, Shenjie Li, Yuting Gao, Wenxiong Tang, Zunjing Liu

**Affiliations:** ^1^Beijing University of Chinese Medicine, Beijing, China; ^2^Department of Neurology, Peking University People’s Hospital, Beijing, China; ^3^Department of Neurology, China-Japan Friendship Hospital, Beijing, China

**Keywords:** albumin, alkaline phosphatase, APAR, ischemic stroke, mortality, biomarker

## Abstract

**Background:**

Recent studies have shown that alkaline phosphatase to albumin ratio (APAR) is a prognostic biomarker for coronary heart disease and cancer. However, the effect of APAR on the prognosis of ischemic stroke (IS) remains unclear. We aimed to assess the association of APAR with all-cause mortality in critically ill patients with IS.

**Methods:**

Critically ill patients with IS were identified from the Medical Information Mart for Intensive Care-IV (MIMIC-IV) Version 3.0 database, and classified into quartiles based on APAR index levels. Clinical outcomes included all-cause mortality at 28-days, 90-days and 365-days after admission. Cox proportional hazards regression analysis and restricted cubic spline method were used to clarify the relationship between APAR index and clinical outcomes in critically ill patients with IS.

**Results:**

A total of 1,690 critically ill patients with IS were selected from the MIMIC-IV database. Multivariate Cox proportional hazard analysis showed that increased APAR index was significantly associated with all-cause mortality. After adjusting for potential confounding factors, patients with higher APAR (Q4: 1.524–2.794) had significantly increased all-cause mortality at 28-days, 90-days, and 365-days after admission (HR 2.05, 95%CI 1.47–2.86, *p* = 0; HR 2.09, 95%CI 1.53–2.85, *p* = 0; HR 2.11, 95%CI 1.55–2.87, *p* = 0). APAR had a linear relationship with 28-days and 365-days mortality (*P* for non-linearity: 0.098 and 0.051), but a nonlinear relationship with 90-days mortality (*P* for non-linearity: 0.042). Subgroup analyses further revealed that higher APAR was also associated with increased long-term mortality in IS patients without hypertension, DM, cardiovascular disease, liver disease or CKD. In addition, we did not observe any interaction between subgroup variables and APAR.

**Conclusion:**

A higher APAR index was significantly associated with increased all-cause mortality at 28-days, 90-days and 365-days after admission for critically ill patients with IS. The APAR index may help identify patients with IS at high risk of all-cause death.

## Introduction

Stroke is a serious global public health issue, characterized by high incidence, high disability rates, and high mortality. Stroke is the second-leading cause of death and the third-leading cause of death and disability combined worldwide in 2019 (1). Ischemic stroke (IS) is often associated with multiple complications, such as cerebral edema, epilepsy, pneumonia, and deep vein thrombosis, which are particularly likely to occur in high-risk groups such as the elderly (2–5). Despite advancements in treatment, such as intravenous thrombolysis and mechanical thrombectomy (6, 7), the risk of poor outcomes in IS patients still remains high, especially in critically ill patients. Therefore, it is crucial to identify prognostic factors associated with adverse outcomes in patients with IS.

In the prognosis assessment of IS, traditional biomarkers and scoring systems such as NIHSS (National Institutes of Health Stroke Scale), mRS (Modified Rankin Scale), CRP (C-reactive protein) and NLR (neutrophil to lymphocyte ratio) have been widely studied and applied (8–10). In recent years, however, novel biomarkers based on routine calculations of peripheral blood, such as alkaline phosphatase (ALP) and serum albumin (ALB), have also shown potential prognostic value. ALP plays an important role in regulating inflammatory processes and mineral metabolism, and may promote atherosclerosis by enhancing vascular calcification (11). Previous studies have found that ALP levels are associated with the severity and prognosis of stroke patients (12, 13). In addition, as an essential protein with anti-inflammatory, antioxidant and antithrombotic properties, ALB levels are also closely related to cardiovascular disease and stroke prognosis (14, 15).

In recent years, the ratio of ALP to ALB (APAR), as a novel indicator of inflammation and nutritional status, has been shown to be a prognostic biomarker for a variety of diseases, such as cancer and coronary heart disease (16, 17). Compared with ALP or ALB alone, APAR provides a more complete picture of the inflammatory and nutritional status of the body, which may provide better prognostic value. For example, in patients with chronic kidney disease (CKD), APAR has been shown to be independently associated with all-cause mortality and has potential value in predicting cardiovascular events (18). In addition, APAR also showed predictive power for adverse outcomes in patients with coronary heart disease (17). However, there are few studies on the relationship between APAR and IS prognosis. Given the critical role of ALP and ALB in atherosclerosis and vascular disease, APAR, as an indicator of the integration of the two information, may be associated with adverse outcomes of IS through inflammation, oxidative stress, and nutritional status. Therefore, this study aimed to investigate the association between APAR and all-cause mortality in patients with severe IS to further evaluate its potential value as a prognostic biomarker.

## Methods

### Study population

**Figure 1:**
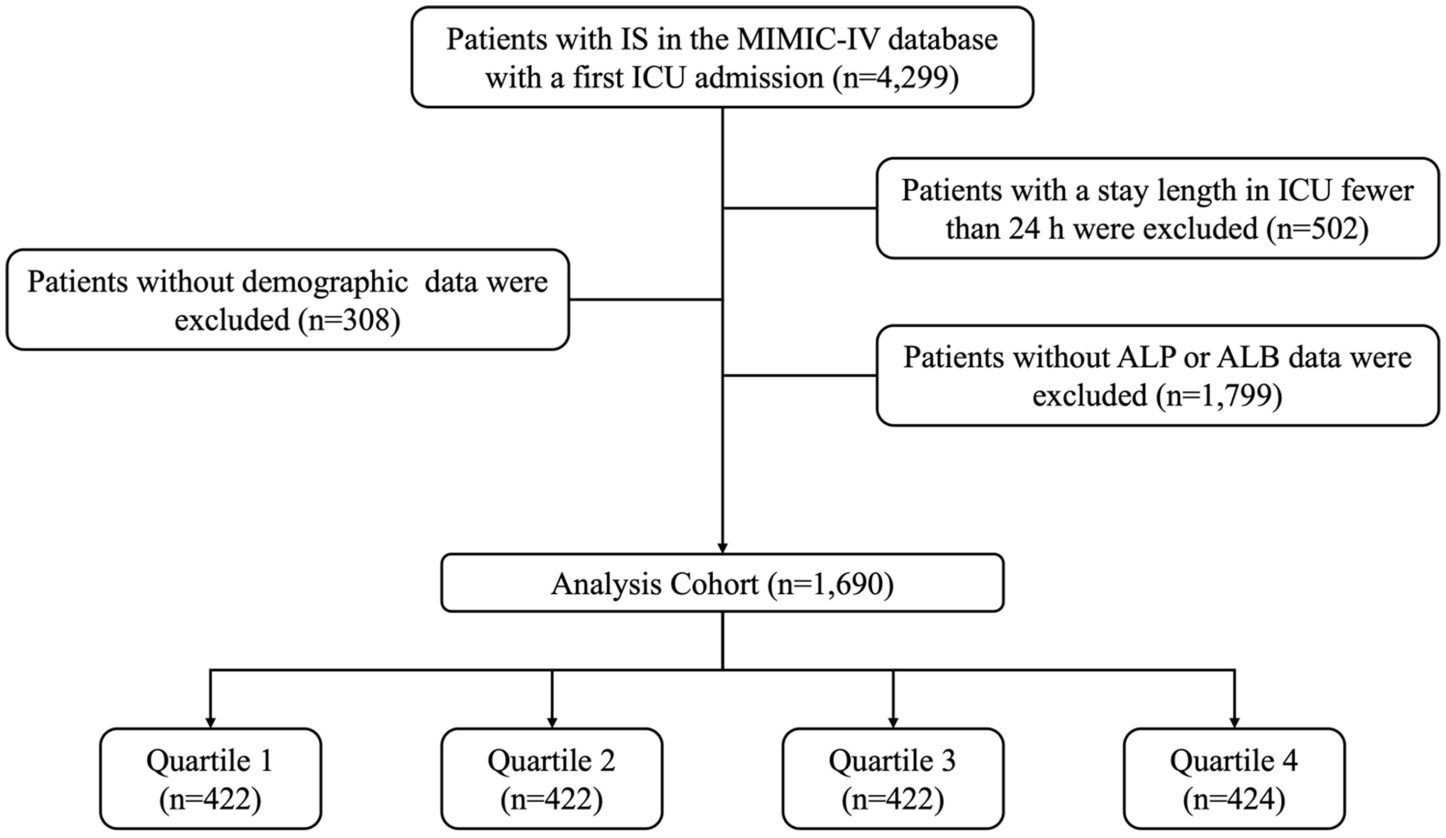
A flowchart illustrating the participant recruitment process. ALB, albumin; ALP, alkaline phosphatase; ICU, intensive care unit; IS, ischemic stroke. MIMIC-IV, medical information mart for intensive care IV.

This study investigated health-related data obtained from the Medical Information Mart for Intensive Care IV (MIMIC-IV) version 3.0 database, which is a large database developed and managed by MIT Computational Physiology Laboratory and consists of extensive medical records of patients admitted to the intensive care unit of Beth Israel Deaconess Medical Center. Data extraction was conducted by one of the authors, Tao Zheng, who met the requirements for accessing the database (ID: 63484863). Based on the International Classification of Diseases, Ninth and Tenth Revision, this study included patients aged 18 years and older diagnosed with ischemic stroke (19). Specifically, the diagnosis of acute ischemic stroke was defined by the ICD-9 codes 43,301, 43,311, 43,321, 43,331, 43,381, 43,391, 43,401, 43,411, and 43,491, as well as the ICD-10 code I63 (20). Exclusion criteria were as follows: (1) patients who stayed in the ICU for less than 24 h; (2) patients who were admitted to the ICU multiple times due to ischemic stroke, with only their first admission data being considered; (3) patients lacking demographic data; (4) patients without sufficient data at admission (including alkaline phosphatase and albumin); (5) patients with abnormal data. Finally, a total of 1,690 patients were enrolled in this study and grouped into four groups based on the quartiles of the APAR index (Figure 1).

### Data collection

The software PostgreSQL (version 13.7.2) alongside Navicat Premium (version 16) was used to extract information with the execution of a Structured Query Language (SQL). The extraction of potential variables could be divided into six principal domains: (1) Demographic characteristics: age and gender; (2) Vital signs: heart rate (HR), systolic blood pressure (SBP), and blood oxygen saturation measured by pulse oxygen saturation (SpO2); (3) Laboratory test results: red blood cell count (RBC), white blood cell count (WBC), hemoglobin (HGB), platelet count (PLT), TC, HDL-C, glucose, serum creatinine (Scr), and serum sodium (Na); (4) Comorbidities: hypertension, diabetes mellitus (DM), hyperlipidemia, cardiovascular disease (CVD), myocardial infarction (MI), heart failure, hepatitis, liver cirrhosis, cancer, and CKD; (5) Clinical severity of illness scores: Sequential Organ Failure Assessment score (SOFA), Acute Physiology Score III (APSIII), Glasgow Coma Scale (GCS), and Charlson comorbidity Index (CCI); (6) Treatment: statins, antihypertensive drugs and mechanical ventilation (MV).

Follow-up began on the date of admission and ended on the date of death. According to previous studies, APAR is calculated by APAR: log10 (ALP/ALB). In addition, we stratified the APAR values of the participants according to the quartile method. To eliminate the missing data influence, covariates with more than 20% missing value were excluded from the analysis. For covariates with less than 20% missing data, we use multiple interpolation techniques to fill in these missing values according to previous research methods (19, 21).

### Clinical outcomes

The primary endpoint of this study was 365-days all-cause mortality after admission. The secondary endpoints included all-cause mortality in 28-days and 90-days all-cause mortality after admission. Survival time was identified by the date of death in the MIMIC-IV database.

### Statistical analysis

Baseline patients’ characteristics were stratified based on APAR values. Continuous variables were expressed as mean ± standard deviation (SD), while classification variables were expressed as frequency (percentage). The normally distributed continuous variables were analyzed by independent sample t-test or analysis of variance (ANOVA). Continuous variables not normally distributed were analyzed by Kruskal-Wallis test. Pearson’s Chi-square test or Fisher exact test was used to compare categorical variables.

Moreover, Kaplan–Meier survival method was used to ascertain the endpoint incidence within groups defined by APAR index. We excluded variables with variance inflation factor (VIF) ≥ 5. The Cox proportional hazards model was used to calculate the hazard ratio (HR) and 95% confidence interval (CI) between APAR index and endpoints, and also adjusted for confounding factors in three models: Model 1 (unadjusted), Model 2 (adjusted for age and sex), and Model 3 (adjusted for age, sex, HR, SBP, SpO2, WBC, RBC, HGB, PLT, Na, glucose, Scr, hypertension, DM, hyperlipidemia, CVD, MI, heart failure, hepatitis, liver cirrhosis, cancer, CKD, statins, antihypertensive drugs, GCS, MV, SOFA, APS III, and CCI). The *p* values for trends were calculated using the quartile level. The APAR index was entered into the models as a continuous variable or a categorical variable (the first quartile of APAR index was taken as the reference group). We also performed a restricted cubic spline regression model with four knots to investigate potential nonlinear relationships between APAR index levels and all-cause mortality. All statistical analyses were performed using R statistical package (R version 4.3). All reported *p* values were two-sided, and *p* < 0.05 were considered statistically significant.

## Results

### Baseline characteristics

Our study ultimately included 1,690 critically ill patients with IS, who were divided into four groups based on the APAR index quartile (Q1: 0.449–1.243; Q2: 1.243–1.36; Q3: 1.361–1.524; Q4: 1.524–2.794) (Table 1). The mean age of the patients was 68.69 ± 15.7 years, and there were 862 (51.01%) male patients. Critically ill patients with IS in the high APAR group exhibited faster HR, lower SBP, higher WBC and ALP levels, lower RBC, HGB, and ALB levels, and higher blood glucose and Scr levels than those in the low APAR group. In addition, they were more likely to have DM, CVD, MI, heart failure, CKD, and cancer, and were more likely to receive MV and antihypertensive treatment, and were less likely to receive statins.

**Table 1:** Characteristics and outcomes of participants categorized by APAR index.

Categories	Overall	Q1	Q2	Q3	Q4	p
	*N* = 1,690	*N* = 422	*N* = 422	*N* = 422	*N* = 424	
Age, years	68.69 ± 15.7	66.54 ± 16.92	68.8 ± 15.58	70.29 ± 14.76	69.12 ± 15.26	0.027
Sex (%)						0.366
F	828 (48.99)	193 (45.73)	214 (50.71)	216 (51.18)	205 (48.35)	
M	862 (51.01)	229 (54.27)	208 (49.29)	206 (48.82)	219 (51.65)	
HR, beats/min	86.71 ± 19.84	82.76 ± 17.77	85.17 ± 19.8	87.73 ± 20.13	91.16 ± 20.63	0
SpO2(%)	97.16 ± 3.37	97.62 ± 2.73	97.31 ± 3.17	96.89 ± 3.69	96.83 ± 3.75	0.011
SBP,mmHg	134.34 ± 27.07	134.73 ± 26.13	137.63 ± 27.09	136.94 ± 26.88	128.06 ± 27.2	0
WBC, K/uL	12.35 ± 9.85	10.66 ± 4.88	11.93 ± 5.56	12.23 ± 13.69	14.57 ± 11.77	0
**RBC**, K/u	3.86 ± 0.82	3.99 ± 0.8	3.99 ± 0.79	3.88 ± 0.79	3.6 ± 0.84	0
**HGB**, g/dL	11.45 ± 2.44	11.92 ± 2.49	11.84 ± 2.37	11.48 ± 2.32	10.55 ± 2.36	0
PLT, K/uL	213.79 ± 104.27	207.86 ± 90.01	220.95 ± 90.44	218.23 ± 97.91	208.15 ± 132.4	0
ALP, IU/L	96.37 ± 78.22	50.92 ± 11.97	69.23 ± 11.41	87.37 ± 16.77	177.58 ± 119.96	0
ALB, g/dL	3.27 ± 0.65	3.61 ± 0.57	3.44 ± 0.53	3.21 ± 0.58	2.82 ± 0.62	0
Glucose, mg/dL	148.94 ± 70.23	137.56 ± 56	147.32 ± 65.29	151.58 ± 74.75	159.25 ± 80.75	0.002
Na, mmol/L	139.16 ± 4.89	139.46 ± 4.19	139.42 ± 4.44	138.96 ± 4.79	138.79 ± 5.94	0.026
Scr, mg/dl	1.37 ± 1.39	1.09 ± 0.91	1.23 ± 1.05	1.43 ± 1.7	1.7 ± 1.63	0
SOFA	4.72 ± 3.56	4.05 ± 3.25	3.97 ± 3.03	4.7 ± 3.48	6.17 ± 3.98	0
APS III	45.66 ± 21.4	40.32 ± 19.06	41.59 ± 18.52	45.68 ± 20.74	55.02 ± 23.76	0
GCS	12.58 ± 3.24	12.69 ± 3.05	12.89 ± 2.88	12.39 ± 3.25	12.34 ± 3.69	0.137
CCI	6.59 ± 2.93	5.75 ± 2.84	6.3 ± 2.66	6.78 ± 2.8	7.51 ± 3.11	0
Hypertension (%)						<0.001
0	859 (50.83)	180 (42.65)	187 (44.31)	219 (51.90)	273 (64.39)	
1	831 (49.17)	242 (57.35)	235 (55.69)	203 (48.10)	151 (35.61)	
DM (%)						<0.001
0	1,127 (66.69)	329 (77.96)	292 (69.19)	264 (62.56)	242 (57.08)	
1	563 (33.31)	93 (22.04)	130 (30.81)	158 (37.44)	182 (42.92)	
Hyperlipidemia (%)						0.089
0	954 (56.45)	237 (56.16)	234 (55.45)	223 (52.84)	260 (61.32)	
1	736 (43.55)	185 (43.84)	188 (44.55)	199 (47.16)	164 (38.68)	
CVD (%)						<0.001
0	1,107 (65.50)	314 (74.41)	294 (69.67)	269 (63.74)	230 (54.25)	
1	583 (34.50)	108 (25.59)	128 (30.33)	153 (36.26)	194 (45.75)	
MI (%)						<0.001
0	1,494 (88.40)	392 (92.89)	390 (92.42)	373 (88.39)	339 (79.95)	
1	196 (11.60)	30 (7.11)	32 (7.58)	49 (11.61)	85 (20.05)	
Heart failure (%)						<0.001
0	1,256 (74.32)	362 (85.78)	336 (79.62)	293 (69.43)	265 (62.50)	
1	434 (25.68)	60 (14.22)	86 (20.38)	129 (30.57)	159 (37.50)	
CKD (%)						<0.001
0	1,386 (82.01)	366 (86.73)	359 (85.07)	345 (81.75)	316 (74.53)	
1	304 (17.99)	56 (13.27)	63 (14.93)	77 (18.25)	108 (25.47)	
Cancer (%)						0.158
0	1,462 (86.51)	369 (87.44)	369 (87.44)	371 (87.91)	353 (83.25)	
1	228 (13.49)	53 (12.56)	53 (12.56)	51 (12.09)	71 (16.75)	
Hepatitis (%)						0
0	1,649 (97.57)	419 (99.29)	416 (98.58)	414 (98.10)	400 (94.34)	
1	41 (2.43)	3 (0.71)	6 (1.42)	8 (1.90)	24 (5.66)	
Liver cirrhosis (%)						0
0	1,626 (96.21)	420 (99.53)	416 (98.58)	410 (97.16)	380 (89.62)	
1	64 (3.79)	2 (0.47)	6 (1.42)	12 (2.84)	44 (10.38)	
MV (%)						<0.001
0	447 (26.45)	140 (33.18)	117 (27.73)	107 (25.36)	83 (19.58)	
1	1,243 (73.55)	282 (66.82)	305 (72.27)	315 (74.64)	341 (80.42)	
Statins (%)						0.029
0	1,394 (82.49)	353 (83.65)	333 (78.91)	342 (81.04)	366 (86.32)	
1	296 (17.51)	69 (16.35)	89 (21.09)	80 (18.96)	58 (13.68)	
Antihypertensive (%)						<0.001
0	541 (32.01)	174 (41.23)	140 (33.18)	122 (28.91)	105 (24.76)	
1	1,149 (67.99)	248 (58.77)	282 (66.82)	300 (71.09)	319 (75.24)	

HR, Heart rate; SpO2, blood oxygen saturation measured by pulse oxygen saturation; SBP, systolic blood pressure; WBC, white blood cell count; RBC, red blood cell count; HGB, hemoglobin; PLT, platelet count; ALB, albumin; ALP, alkaline phosphatase; Na, serum sodium; Scr, hypertension, DM, diabetes mellitus; CVD, cardiovascular disease; MI, myocardial infarction; CKD, chronic kidney disease; MV, mechanical ventilation; APS III, acute physiology score III; CCI, Charlson comorbidity Index; GCS, Glasgow coma scale; SOFA, sequential organ failure assessment score.

### Clinical outcomes

**Figure 2:**
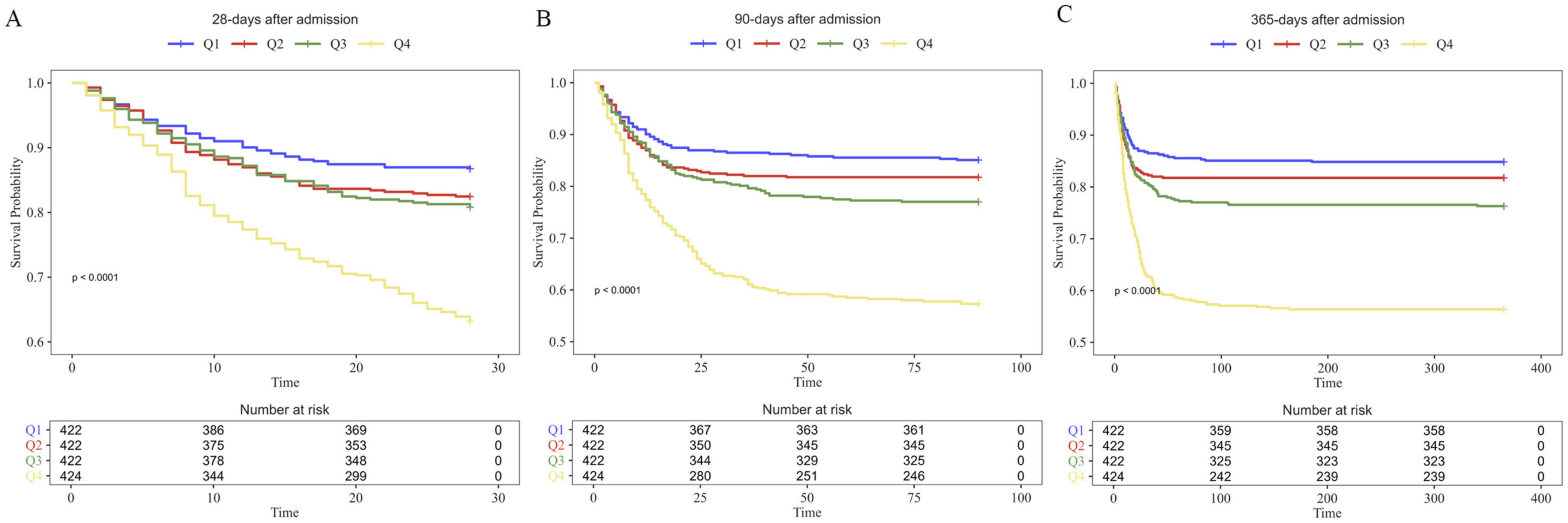
Kaplan–Meier survival analysis curves for all-cause mortality.

The Kaplan–Meier survival analysis curves were employed to analyze incidence of primary and secondary outcomes among groups based on APAR quartiles. Patients with APAR ranged from 0.449 to 1.243 (Q1) had a lowest risk of death in 28-days, at 90-days and 365-days after admission (A: log-rank *p* < 0.0001; B: log-rank *p* < 0.0001; C: log-rank *p* < 0.0001) (Figure 2).

Cox proportional risk analysis was used to analyze the association between APAR and mortality. When the APAR was a continuous variable, the findings indicated that APAR was a significant predictor of mortality in 28-days in the three models (Model 1: HR 5.37, 95% CI 3.48–8.31, *p* = 0; Model 2: HR 5.38, 95%CI 3.48–8.36, *p* = 0; Model 3: HR 2.39, 95% CI 1.44–3.97, *p* < 0.001), 90-days mortality in the three models (Model 1: HR 7.01, 95% CI 4.58–10.83, *p* = 0; Model 2: HR 7.04, 95%CI 4.58–10.9, *p* = 0; Model 3: HR 2.92, 95% CI 1.78–4.8, *p* = 0), and 365-days in the three models (Model 1: HR 7.78, 95% CI 5.07–12.04, *p* = 0; Model 2: HR 7.81, 95% CI 5.08–12.12, *p* = 0; Model 3: HR 3.25, 95%CI 1.99–5.33, *p* = 0). When APAR was a nominal variable, patients in the Quartile 4 had a significantly increased risk of death in 28-days (HR 2.05, 95%CI 1.47–2.86, *p* = 0), 90-days (HR 2.09, 95%CI 1.53–2.85, *p* = 0), and 365-days (HR 2.11, 95%CI 1.55–2.87, *p* = 0) mortality in the fully adjusted model 3 (Table 2).

**Table 2:** The relationships between APAR and all-cause mortality.

Categories	Model 1			Model 2			Model 3		
	HR (95% CI)	*p*-value	*P* for trend	HR (95% CI)	*P*-value	*P* for trend	HR (95% CI)	*P*-value	*P* for trend
28-days after admission									
Continuous variable per unit	5.37 (3.48, 8.31)	0		5.38 (3.48, 8.36)	0		2.39 (1.44, 3.97)	<0.001	
Quartile			0			0			0
Q1 (*N* = 422)	Ref			Ref			Ref		
Q2 (*N* = 422)	1.35 (0.96,1.92)	0.088		1.34 (0.94, 1.89)	0.103		1.28 (0.89, 1.82)	0.179	
Q3 (*N* = 422)	1.48 (1.06, 2.09)	0.023		1.44 (1.03,2.03)	0.036		1.25 (0.88, 1.78)	0.215	
Q4 (*N* = 424)	3.1 (2.28, 4.2)	0		3.03 (2.23, 4.12)	0		2.05 (1.47, 2.86)	0	
90-days after admission									
Continuous variable per unit	7.01 (4.58, 10.83)	0		7.04 (4.58, 10.9)	0		2.92 (1.78, 4.8)	0	
Quartile			0			0			0
Q1 (*N* = 422)	Ref			Ref			Ref		
Q2 (*N* = 422)	1.26 (0.9, 1.75)	0.18		1.24 (0.89, 1.74)	0.2		1.16 (0.83, 1.63)	0.383	
Q3 (*N* = 422)	1.59 (1.16, 2.19)	0.004		1.56 (1.11, 2.14)	0.006		1.31 (0.95, 1.82)	0.104	
Q4 (N = 424)	3.29 (2.47, 4.39)	0		3.23 (2.43, 4.31)	0		2.09 (1.53, 2.85)	0	
365-days after admission									
Continuous variable per unit	7.78 (5.07, 12.04)	0		7.81 (5.08, 12.12)	0		3.25 (1.99, 5.33)	0	
Quartile			0			0			0
Q1 (*N* = 422)	Ref			Ref			Ref		
Q2 (*N* = 422)	1.24 (0.89, 1.72)	0.209		1.22 (0.88, 1.71)	0.232		1.15 (0.82, 1.62)	0.405	
Q3 (*N* = 422)	1.62 (1.18, 2.22)	0.003		1.58 (1.16, 2.17)	0.004		1.34 (0.97, 1.85)	0.078	
Q4 (*N* = 424)	3.33 (2.5, 4.42)	0		3.27 (2.46, 4.35)	0		2.11 (1.55, 2.87)	0	

Model 1 (unadjusted), Model 2 (adjusted for age and sex), and Model 3 (adjusted for age, sex, HR, SBP, SpO2, WBC, RBC, HGB, PLT, Na, glucose, Scr, hypertension, DM, hyperlipidemia, CVD, MI, heart failure, hepatitis, liver cirrhosis, cancer, CKD, statins, antihypertensive drugs, GCS, MV, SOFA, APS III, and CCI). HR, hazard ratio; CI, confidence interval.

**Figure 3:**
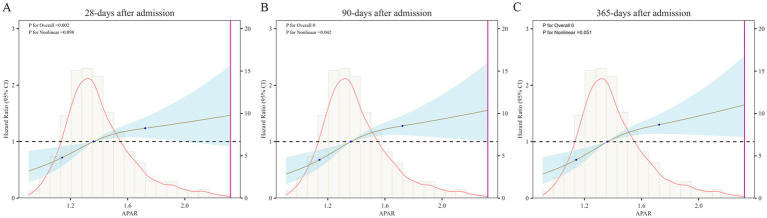
Restricted cubic spline regression analysis for all-cause mortality. The heavy central lines represent the estimated adjusted hazard ratios, with blue bands denoting 95% confidence intervals. APAR, alkaline phosphatase to albumin ratio.

Furthermore, we observed the APAR had a linear relationship with 28-days and 365-days mortality (*P* for non-linearity: 0.098, *P* for non-linearity: 0.051, respectively), but a nonlinear relationship with 90-days mortality (*P* for non-linearity: 0.042) (Figure 3).

### Subgroup analysis

**Figure 4:**
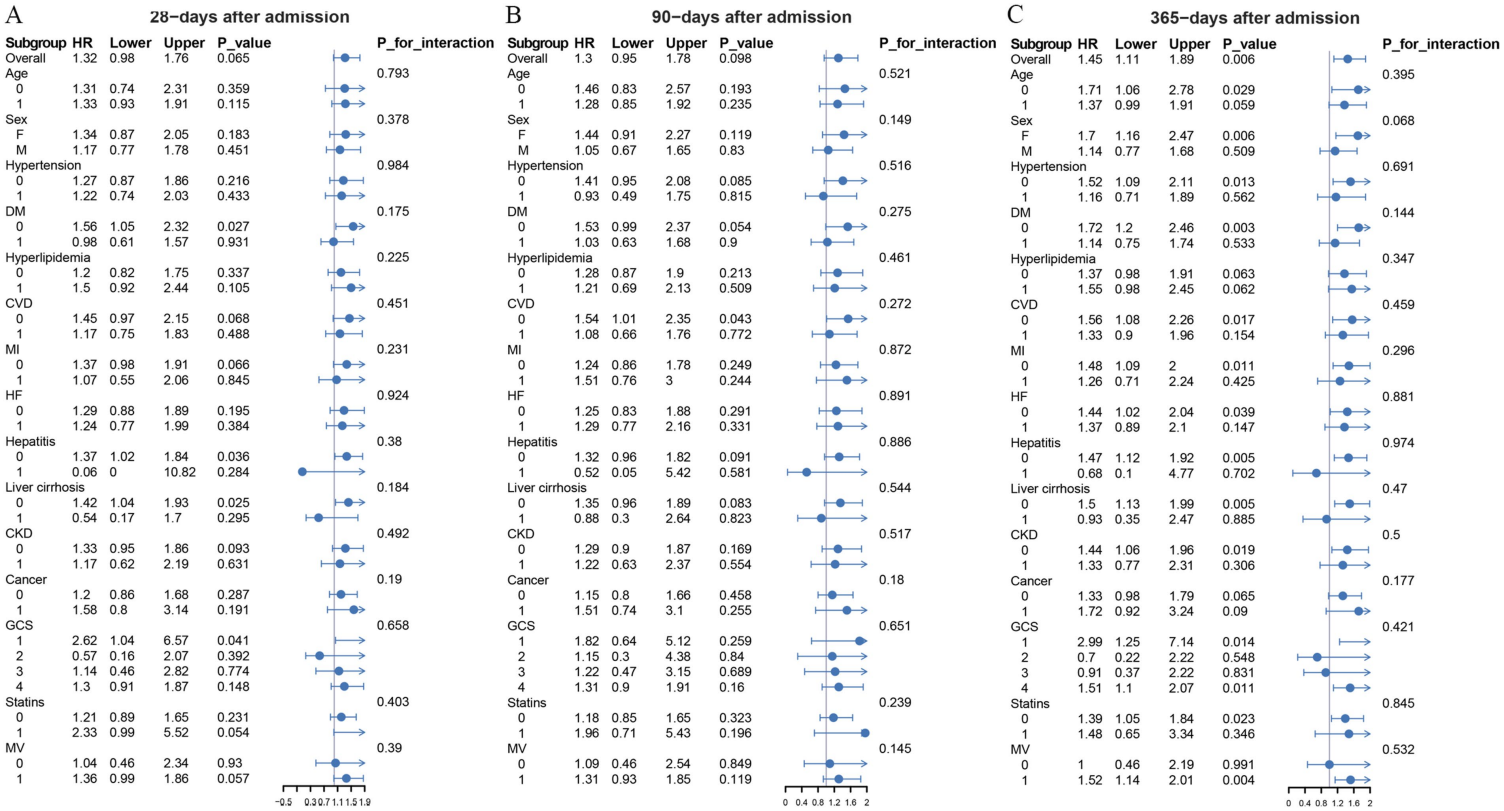
Forest plots of hazard ratios for mortality in different subgroups. GCS grades: grade 1, 3–5 scores; grade 2, 6–8 scores; grade 3 9–12 scores; grade 4, 13–15 scores. CI, confidence interval; HR, hazard ratio; DM, diabetes mellitus; CVD, cardiovascular disease; MI, myocardial infarction; HF, heart failure; CKD, chronic kidney disease; GCS, Glasgow coma scale; MV, mechanical ventilation.

The prognostic value of APAR index for all-cause mortality was further analyzed across various subgroups, including age, sex, hypertension, DM, hyperlipidemia, heart failure, MI, CVD, hepatitis, liver cirrhosis, CKD, cancer, statins and GCS grades. The turning points identified by threshold effect analysis were 1.7404 in 28-days, 1.8472 in 90-days and 1.7404 in 365-days after admission. The APAR index was significantly associated with higher risk of death in female (HR 1.71, 95%CI 1.06–2.78, *p* = 0.029) at 365-days after admission. Higher APAR was also associated with increased 365-days after admission mortality in IS patients without hypertension, DM, cardiovascular disease, liver disease, or CKD (Figure 4). For example, it was significantly associated with higher risk of death in non-hypertension group (HR 1.52, 95%CI 1.09–2.11, *p* = 0.013), non-DM group (HR 1.72, 95%CI 1.2–2.46, *p* = 0.003), non-CVD group (HR 1.56, 95%CI 1.08–2.26, *p* = 0.017) at 365-days after admission. In addition, we did not observe any interaction between subgroup variables and APAR.

## Discussion

This study is the first to evaluate the role of APAR index in predicting short-and long-term outcomes in critically ill patients with IS. The results showed that, after controlling for potential confounding factors, higher APAR was significantly associated with increased all-cause mortality at 28-days, 90-days, and 365-days after admission. APAR had a linear relationship with 28-days and 365-days mortality (*P* for non-linearity: 0.098, *P* for non-linearity: 0.051, respectively), but a nonlinear relationship with 90-days mortality (*P* for non-linearity = 0.042). Subgroup analyses further revealed that higher APAR was also associated with increased long-term mortality in IS patients without hypertension, DM, cardiovascular disease, liver disease, or CKD. In addition, we did not observe any interaction between subgroup variables and APAR. Therefore, APAR has the potential to be a useful tool for clinicians in the decision-making process and may be recognized as an independent risk factor for critically ill patients with IS.

APAR is a biomarker that combines ALP and ALB, which has been gradually paid attention in medical research in recent years, especially in the prognostic evaluation of some diseases. Abnormal serum ALP levels are associated with a variety of neurological diseases, such as small cerebral vascular disease, Alzheimer’s disease, traumatic brain injury and so on. One study found that after adjusting for cardiovascular risk factors, subjects with ALP ≥ 195 IU/L had a significantly increased risk of asymptomatic lacunar infarction (SLI) and moderate to severe white matter hypersignaling (MS-cWMH) compared with ALP ≤ 155 IU/L (22). Vardy et al. found that ALP was elevated in both brain and plasma in Alzheimer’s patients and negatively correlated with cognitive function (23). In addition, serum ALP is also significantly increased in patients with traumatic brain injury (24). Many studies have found that ALP is closely related to stroke, and it has been considered as a potential diagnostic and prognostic indicator of stroke. Studies have shown that higher serum ALP levels, even if it is in the normal range, are significantly associated with a higher risk of first stroke in Chinese adults with hypertension (25). A Japanese study also found that for non-drinkers, higher ALP levels were associated with an increased risk of ischemic stroke in men and hemorrhagic stroke in women (26). In addition, the results of a meta-analysis in conjunction with the Mendelian Randomization study also confirmed that a higher ALP significantly increased the risk of stroke (27). ALP is not only associated with the risk of stroke, but also with the prognosis after stroke. A higher ALP has been found to significantly increase the 3-month all-cause mortality in patients with acute stroke (28). It has also been found that higher ALP significantly increases in-hospital mortality in patients with acute ischemic stroke (29). ALB is the most abundant circulating protein in blood, which is protective in several diseases, including CHD, heart failure, hypertension, atrial fibrillation, and peripheral artery disease (14). Some observational studies and meta-analyses suggested that hypoalbuminemia was a strong prognosticator of increased all-cause and cardiovascular mortality (30–32). In several studies, ALB was involved as a key factor for stroke risk and prognosis. ALB may serve as a correction for certain indicators to better study the relationship between these indicators and stroke. For example, numerous studies have shown that ratios such as neutrophil-to-albumin ratio (33), fibrinogen-to-albumin ratio (34), C-reactive protein to albumin ratio (35, 36) and High serum lactate dehydrogenase to albumin ratio (37) are closely related to stroke. Some studies showed that low ALB levels are associated with poor prognosis in stroke, including all-cause death, heart failure, atrial fibrillation, ventricular arrhythmias, and myocardial infarction (38).

In Model 3, APAR was positively associated with short-and long-term mortality in patients with IS, even after we included 29 covariates in our analysis, including adjustments for biochemical markers and comorbidities. In addition, subgroup analysis showed that no significant interaction was found between any particular treatment modality or comorbidities and APAR. Therefore, APAR may be a relatively independent prognostic indicator with a certain universality in predicting mortality in patients with ischemic stroke. The prognostic value of APAR in ischemic stroke may reflect unique pathophysiological mechanisms. The increase of ALP level may be closely related to the breakdown of blood–brain barrier (BBB) after IS. Studies have shown that ALP is highly expressed in cerebrovascular endothelial cells and plays an important role in the permeability, maintenance and integrity of the BBB and the transport of proteins through the barrier (39, 40). Increased ALP levels may reflect endothelial dysfunction and increased blood–brain barrier permeability, leading to increased cerebral edema and neurological deterioration. After IS, the inflammatory response of the body is activated rapidly, and the levels of inflammatory factors such as TNF-*α* and IL-6 are significantly increased (41). ALP may act as a regulator of inflammatory mediators during inflammation, and its elevated level may reflect the excessive neuroinflammatory response after stroke. At the same time, low albumin levels may impair its antioxidant and anti-inflammatory effects, further exacerbating neuroinflammatory damage (42). Oxidative stress plays a key role in secondary brain injury after IS. Elevated ALP levels may be associated with increased production of reactive oxygen species (43), while low albumin levels impair its ability to scavenge free radicals. This imbalance may lead to increased neuronal damage and affect the prognosis of patients. In addition, low albumin levels may reflect the acute phase reaction and worsening nutritional status after IS. Stroke-related dysphagia, metabolic alterations, and increased catabolism can lead to protein-energy malnutrition, which not only affects nerve repair, but may also increase the risk of complications. For example, ALB is inversely associated with hemorrhagic conversion in patients with acute ischemic stroke (44). However, the underlying biological mechanism of increased APAR levels and poor prognosis in patients with IS remains to be explored.

To further evaluate the prognostic value of APAR in ischemic stroke, we performed subgroup analyses that included coronary heart disease, myocardial infarction, heart failure, hepatitis, liver cirrhosis, and cancer. In previous studies, APAR has been confirmed to be closely related to the occurrence and progression of coronary heart disease, and is considered as a potential biomarker. However, in the subgroup analysis of this study, we found that the association between APAR and mortality risk has specific population characteristics. Specifically, only in patients without coronary heart disease, those with higher APAR levels had a significantly increased risk of death at 90 and 365 days after admission, while this association was not seen in patients with coronary heart disease. In addition, we did not observe a significant interaction between APAR and coronary heart disease. We also observed this phenomenon in patients with no prior myocardial infarction or heart failure at 365 days after admission. ALP is a marker of vascular calcification, which promotes the process of vascular calcification through several mechanisms, including catalyzing pyrophosphate hydrolysis, promoting vascular smooth muscle cell calcification, and participating in inflammation (45–47). Moreover, low ALB levels can promote pulmonary edema and fluid retention, leading to heart failure (48). In patients without vascular disease, a higher APAR may imply a significantly increased risk of cardiovascular disease in these patients, resulting in increased mortality.

As both ALP and ALB are key liver function indicators, we specifically examined the effect of APAR on IS patients with hepatitis or cirrhosis in our subgroup analysis. However, the results showed that a higher APAR was associated with significantly increased mortality in patients with IS during the observation period of 28 and 365 days after admission for those without hepatitis or cirrhosis. In patients without liver disease, the synthetic function of the liver is relatively normal, and the elevated level of ALP can better reflect the activation of systemic inflammatory response, while the level of ALB can better reflect the overall nutritional and metabolic status of the body. Higher APAR may indicate that the body lacks adequate anti-inflammatory and repair capacity after IS, thus increasing the risk of death. In addition, our subgroup analysis did not find an association between higher APAR and an increased risk of death in IS patients with co-existing hepatitis or cirrhosis. Moreover, in a subgroup analysis of cancer patients, we did not find a significant association between higher APAR and an increased risk of death in ischemic stroke patients with cancer. During long-term follow-up, we observed that higher APAR was associated with significantly increased mortality in ischemic stroke patients without hypertension, diabetes, or CKD. ALP can lead to increased vascular stiffness and decreased vascular compliance. A higher APAR significantly increases the risk of high blood pressure (49). It has also been found that higher serum ALP is significantly associated with an increased risk of new-onset diabetes (50). And higher ALP is also closely related to CKD and uremia (11, 45). In addition, although no significant association between APAR and risk of death was found in IS patients with hepatitis or cirrhosis and in subgroups of cancer patients, higher APAR was associated with a significantly increased risk of death in specific high-risk patient groups (e.g., GCS grade 1, non-statin users, and patients receiving mechanical ventilation). This suggests that APAR may have potential applications in identifying high-risk patients.

Although this study revealed the APAR index as an important independent predictor of all-cause mortality in patients with severe IS after adjusting for multiple confounders, some limitations remain. First, retrospective study design limits the certainty of causation and makes it difficult to establish a direct causal link between APAR and mortality. Second, despite our efforts to adjust for known confounders, given limitations in the study design, there may still be confounders that are not identified or are not adequately adjusted for, potentially affecting clinical outcomes. In addition, although we included a large amount of patient data, the integrity and accuracy of the data may be affected by the original data collection standards and methods. Finally, due to the lack of external validation of independent cohorts, the generalisability and robustness of the findings need to be further confirmed.

## Conclusion

A higher APAR index was significantly associated with increased all-cause mortality at 28, 90, and 365 days after admission for critically ill patients with IS. The APAR index may help identify patients with IS at high risk of all-cause death.

## Data Availability

The datasets presented in this study can be found in online repositories. The names of the repository/repositories and accession number(s) can be found in the article/supplementary material.

## Glossary

HR: Heart rate
SpO2: Blood oxygen saturation measured by pulse oxygen saturation
SBP: Systolic blood pressure
WBC: White blood cell count
RBC: Red blood cell count
HGB: Hemoglobin
PLT: Platelet count
ALB: Albumin
ALP: Alkaline phosphatase
Na: Serum sodium
Scr: Hypertension
DM: Diabetes mellitus
CVD: Cardiovascular disease
MI: Myocardial infarction
CKD: Chronic kidney disease
MV: Mechanical ventilation
APS III: Acute Physiology Score III
CCI: Charlson comorbidity Index
GCS: Glasgow coma scale
SOFA: Sequential Organ Failure Assessment score
